# Disulfiram Improves the Anti-PD-1 Therapy Efficacy by Regulating PD-L1 Expression *via* Epigenetically Reactivation of IRF7 in Triple Negative Breast Cancer

**DOI:** 10.3389/fonc.2021.734853

**Published:** 2021-11-10

**Authors:** Xin Zheng, Zijian Liu, Mi Mi, Qiuyue Wen, Gang Wu, Liling Zhang

**Affiliations:** Cancer Center, Union Hospital, Tongji Medical College, Huazhong University of Science and Technology, Wuhan, China

**Keywords:** disulfiram, triple negative breast cancer, immune checkpoint blockade, PD-L1, DNMT1, IRF7

## Abstract

Immune checkpoint blockade (ICB), particularly programmed death 1 (PD-1) and its ligand (PD-L1), has shown considerable clinical benefits in patients with various cancers. Many studies show that PD-L1 expression may be biomarkers to help select responders for anti-PD-1 treatment. Therefore, it is necessary to elucidate the molecular mechanisms that control PD-L1 expression. As a potential chemosensitizer and anticancer drug, disulfiram (DSF) kills tumor cells *via* regulating multiple signaling pathways and transcription factors. However, its effect on tumor immune microenvironment (TIME) remains unclear. Here, we showed that DSF increased PD-L1 expression in triple negative breast cancer (TNBC) cells. Through bioinformatics analysis, we found that DNMT1 was highly expressed in TNBC tissue and PD-L1 was negatively correlated with IRF7 expression. DSF reduced DNMT1 expression and activity, and hypomethylated IRF7 promoter region resulting in upregulation of IRF7. Furthermore, we found DSF enhanced PD-L1 expression *via* DNMT1-mediated IRF7 hypomethylation. In *in vivo* experiments, DSF significantly improved the response to anti-PD-1 antibody (Ab) in 4T1 breast cancer mouse model. Immunohistochemistry staining showed that granzyme B+ and CD8+ T cells in the tumor tissues were significantly increased in the combination group. By analyzing the results of the tumor tissue RNA sequencing, four immune-associated pathways were significantly enriched in the DSF joint anti-PD-1 Ab group. In conclusion, we found that DSF could upregulate PD-L1 in TNBC cells and elucidated its mechanism. Our findings revealed that the combination of DSF and anti-PD-1 Ab could activate TIME to show much better antitumor efficacy than monotherapy.

## Introduction

Breast cancer (BC) is one of the most frequent diseases and the leading cause of cancer death among females (1). Although molecular targeted therapy such as trastuzumab, pertuzumab, lapatinib has achieved good curative effects on human epidermal growth factor receptor-2 (HER-2)-positive BC, there is limited efficacious treatment options for triple negative BC (TNBC) (2). TNBC (progesterone receptor, estrogen receptor, and HER-2 negative BC) is an aggressive subtype of BC that accounts for 15–20% of BC patients, with very poor prognosis (3). There is an urgent need to explore novel therapeutic strategies to improve the clinical outcomes of TNBC patients.

Over the last decades, immunotherapy, especially immune checkpoint inhibitors, has shown considerable clinical benefits in patients with various cancers (4–6). T cell surface expressing PD-1 plays a vital role in negatively regulating the functions of antitumor T cell effector upon interacting with its PD-L1 expressed on tumor cell surface. In the tumor microenvironment (TME), inducing PD-L1 expression can lead to PD-1-mediated T cell exhaustion, thus suppressing the antitumor cytotoxic T cell response (7). Such negative interaction can be inhibited by anti-PD-1/anti-PD-L1 antibodies (Abs). PD-1/PD-L1 checkpoint blockades have been approved by the FDA in various cancers, including lung cancer, Hodgkin lymphoma, and BC (8–10). Although pembrolizumab has been approved in TNBC, the overall response rate was only 18.5%, reported in KEYNOTE-012 trial (11). Therefore, improving the therapeutic effect of anti-PD-1 antibody (Ab) in TNBC patients is urgent and valuable.

Numerous studies have identified biomarkers predicting response of anti-PD-1 therapy, and found that tumor mutational load, dense CD8+ T-cell infiltrates, and PD-L1 expression may be biomarkers to help select responders to anti-PD-1 Ab (12–15). In the TME, PD-L1 expression is regulated at epigenetic, transcriptional, and post-transcriptional levels. At epigenetic level, inhibiting DNA methylation upregulates the expression of PD-L1. Several studies reported that DNA methyltransferase (DNMT) inhibitors decitabine and azacytidine could increase PD-L1 expression level and enhance anti-PD-1 Ab therapeutic efficacy in tumor cells (16–18). At transcriptional level, several transcriptional factors, such as NF-κB, STAT3, and interferon regulatory factor 7 (IRF7), are involved in PD-L1 expression (19, 20). IRF7, a master regulator of type I interferon response, can upregulate PD-L1 expression through PD-L1 promoter binding. Recent study reported that hypomethylating IRF7 by decitabine resulted in elevated levels both IRF7 and PD-L1 (21). Restoration of IRF7 can also affect the activation of immune cells, leading to a remodeling of TME (21–23). As such, we may infer that regulation of DNMT1/IRF7/PD-L1 could sensitize TNBC cells through inducing PD-L1 by DNMT1 inhibitors.

Besides decitabine and azacytidine are DNMT inhibitors, which have won FDA approval for treating myelodysplastic syndromes, disulfiram (DSF) also acts as a DNMT inhibitor. DSF has been employed to treat alcohol use disorders for over sixty years. Increasing evidences indicate that DSF can be repurposed as a novel anti-cancer drug by regulating tumor cell growth, angiogenesis, apoptosis, epithelial-to-mesenchymal transition (EMT) and stemness (24–26). Lin and colleagues reported that DSF monotherapy inhibited DNMT1 catalytical activity and resulted in inhibition of prostate cancer growth (27). However, Dastjerdi and colleagues failed to confirm the DNA demethylation effect of DSF in pancreatic cancer cell line (28). The discrepancy of DSF on DNMT activity perhaps is due to different cancer types or others. Whether DSF has potent DNA demethylation function in TNBC needs to be further explored. Furthermore, Zhou et al. reported that DSF combined with copper upregulated PD-L1 expression by inhibiting PARP1 activity in hepatocellular carcinoma (11). Moreover, a recent study demonstrated that DSF combined with an anti-PD-1 Ab synergistically suppressed tumor growth by targeting FROUNT (also known as NUP85) function and elevated the number of CD8+ T cells in the tumors (29). Accordingly, DSF could enhance the responsiveness to immune checkpoint blockade (ICB) therapy; however, the underlying mechanism remains unclear (30).

DNMT1 plays an essential role in tumorigenesis of TNBC involving suppression of estrogen receptor expression, promotion of epithelial-to-mesenchymal transition (EMT), and induction of stemness in TNBC (31). Although targeting DNMT1 in TNBC by azacytidine did not show sufficient efficacy (32, 33), combination of decitabine and anti-PD-1 Ab may sensitize TNBC patients for anti-PD-1 therapy by inducing the expression of PD-L1, and the regimen is currently investigated in the neoadjuvant setting (NCT02957968). In this study, we explored whether epigenetic regulation associated with DNA methylation could underlie increasing PD-L1 expression by DSF. We found that DSF inhibited DNMT1 activity, thus leading to IRF7 hypomethylation and PD-L1 upregulation in TNBC cell lines. We further observed that co-treatment of DSF and anti-PD-1 Ab increased CD8+ tumor infiltrating lymphocytes (TIL) and enhanced the therapeutic effects of ICB *in vivo*.

## Results

### DSF Inhibits DNMT1 Expression and Activity in TNBC Cells

**Figure 1:**
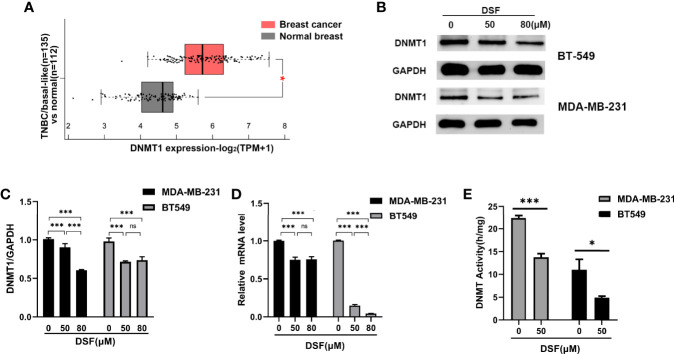
DSF inhibits DNMT1 expression and activity *in vitro*. **(A)** Gene expression analysis of DNMT1 using GEPIA2 database based on the TCGA database. Box plots represent the gene expression level in terms of log2 (TPM+1) in the tumor (TNBC/basal-like subtype, red, n=135) and normal breast tissue (gray, n=112) samples, respectively. **(B)** DNMT1 expression in human BT-549 and MDA-MB-231 cells by Western blotting after treatment with different doses of DSF for 48 h. **(C)** Quantitative analysis of DNMT1 expression after different doses of DSF for 48 h through ImageJ intensity measurements. **(D)** The relative mRNA expression levels of DNMT1 in human MDA-MB-231 cells and BT-549 cells treated with indicated concentration of DSF for 48 h. **(E)** DNMT1 enzyme activity assays in human MDA-MB-231 and BT-549 cells treated with indicated concentration of DSF for 48 h. *p < 0.05, ***p < 0.001. ns, no statistic significance.

The mRNA expression of DNMT1 was remarkably higher in TNBC/basal-like subtype (BLS; n=135) than in normal breast tissue (NBT; n=112; p=3.00×10–36) based on Gene Expression Profiling Interactive Analysis 2 (GEPIA2) in **Figure 1A**. To investigate the DNA demethylation effect of DSF in TNBC cell lines, we tested the protein levels of DNMT1 in human BT-549 and MDA-MB-231 cell lines exposed to DSF at different does for 48 h by western blot. As shown in **Figures 1B, C**, the DNMT1 expression was significantly reduced by DSF. At transcription level, a consistent phenomenon was observed in human MDA-MB-231 cells and BT-549 cells exposed to the indicated concentrations of DSF for 48 h in **Figure 1D**. Further experiments showed that the activity of DNMT was also inhibited by DSF in BT-549 and MDA-MB-231 cells exposed to DSF for 48 h (**Figure 1E**).

### DSF Upregulates IRF7 by Hypomethylating IRF7 in TNBC Cells

**Figure 2:**
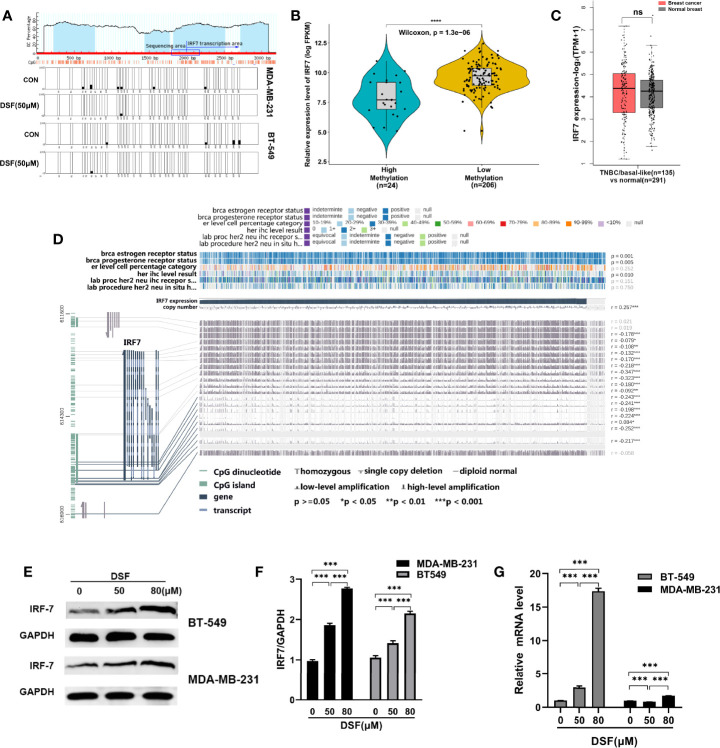
DSF upregulates IRF7 by hypomethylating IRF7. **(A)**The hypomethylation status of IRF7 promoter regions assessed by bisulfite sequencing analysis in the human MDA-MB-231 cells and BT-549 cells treated with DSF (50 μM) for 48 h. **(B)** The negative relationship between the mRNA expression of IRF7 and the methylation level of IRF7. The samples in TCGA are divided into high methylation groups (green, n=24) and low methylation groups (yellow, n= 206). **(C)** Gene expression analysis of IRF7 using GEPIA2 database based on the TCGA and GTEx database. Box plots represent the gene expression level in terms of log2 (TPM+1) in the tumor (TNBC/basal-like subtype, red, n=135) and normal breast tissue (gray, n=291) samples, respectively. **(D)** Visualization of TCGA data for IRF7 expression in 1,217 patients using MEXPRESS. The samples are ordered by their expression value. This view shows the relationship between IRF7 expression and methylation around CpG island and promoter region, clinical features, as well as CNVs. Statistical significance was indicated in the right side. **(E)** IRF7 expression in human BT-549 and MDA-MB-231 cells by Western blotting after treatment with different doses of DSF for 48 h. **(F)** Quantitative analysis of IRF7 expression after different doses of DSF for 48 h through ImageJ intensity measurements. **(G)** The relative mRNA expression levels of IRF7 in human MDA-MB-231 cells and BT-549 cells treated with indicated concentration of DSF for 48 h. ***p < 0.001, ****p < 0.0001. ns, no statistic significance.

We explored whether DSF can affect the methylation level of IRF7 by affecting DNMT1, thereby regulating its expression. First, we analyzed the hypomethylation status of IRF7 promoter regions assessed by bisulfite sequencing analysis in the human MDA-MB-231 cells and BT-549 cells exposed to DSF (**Figure 2A**). To unravel the underlying relationship between the expression and the DNA methylation level of IRF7 in BC, we analyzed data from 1,217 patients in TCGA. The tumor samples are divided into high methylation groups (n=24) and low methylation groups (n= 206). In **Figure 2B**, the violin-box plot revealed that IRF7 mRNA expression was negatively correlated with its DNA methylation level. Gene expression analysis of IRF7 using GEPIA2 database based on the TCGA and GTEx database. Box plots represent the gene expression level in terms of log2 (TPM+1) in the tumor (TNBC/basal-like subtype, red, n=135) and normal breast tissue (gray, n=291) samples, respectively (**Figure 2C**). As presented in **Figure 2D**, the results showed that there was a negative correlation between IRF7 mRNA expression and DNA-methylated CpG islands in the gene body region, especially the promoter region. The immunoblot results revealed that the protein levels of IRF7 were upregulated in human BT549 cells and MDA-MB-231 cells exposed to DSF at different doses for 48 h (**Figures 2E, F**). Next, real-time PCR (RT-PCR) was conducted to determine the mRNA expression of IRF7. Notably, the expression level of IRF7 was remarkably upregulated in human MDA-MB-231 cells and BT-549 cells exposed to the indicated concentration of DSF for 48 h (**Figure 2G**).

### DSF Increases PD-L1 Expression in TNBC Cell Lines

**Figure 3:**
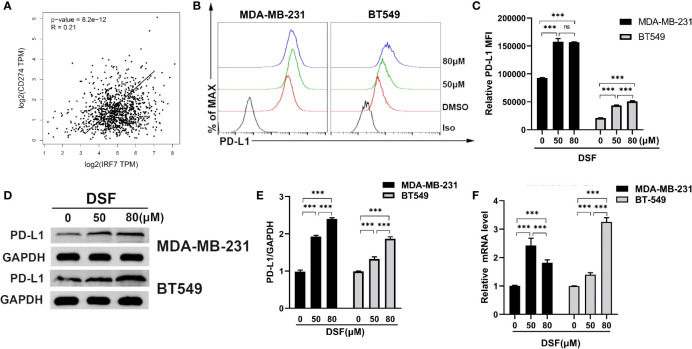
DSF increases PD-L1 expression *in vitro*. **(A)** The positive relationship between the mRNA expression of PD-L1 and IRF-7 in breast cancer. **(B)** Flow cytometry result of surface level of PD-L1 on MDA-MB-231 cell and BT-549 cell treated with different doses of DSF for 48 h. **(C)** The quantification of relative mean fluorescence intensity (MFI) of PD-L1 on MDA-MB-231 cell and BT-549 cell treated with different doses of DSF for 48 h. **(D)** PD-L1 protein expression after DSF treatment. MDA-MB-231 and BT-549 cells were treated with different doses of DSF for 48 h, and PD-L1 protein levels were analyzed by Western blotting. **(E)** Quantitative analysis of PD-L1 expression after different doses of DSF for 48 h through ImageJ intensity measurements. **(F)** The relative mRNA expression levels of PD-L1 in human MDA-MB-231 cells and BT-549 cells treated with indicated concentration of DSF for 48 h. ***p < 0.001. ns, no statistic significance.

Type I interferon regulated by IRFs can induce PD-L1 expression (34). We observed that the mRNA expression of PD-L1 was positively associated with IRF7 in BC patients from TCGA database (R=0.21, p<0.001) in **Figure 3A**. Human BT-549 and MDA -MB-231 cells were exposed to DSF for 48 h at doses 50 and 80 μM. DSF treatments significantly increased surface PD-L1 level and induced a twofold increase in PD-L1 levels. Flow cytometry and immunoblotting showed that PD-L1 protein expression was upregulated in the human MDA-MB-231 and BT549 cells after DSF treatment in **Figures 3B–E**. In human BT-549 cells, RT-PCR analysis further indicated that DSF treatment upregulated the mRNA expression of PD-L1 (**Figure 3F**), suggesting that DSF controls PD-L1 expression at the transcriptional levels. Upregulation of PD-L1 surface expression was found in human BT-549 cell line treated with DSF. Collectively, these findings show that DSF can upregulate the transcription and surface expression of PD-L1 in TNBC cell lines.

### DSF Regulates PD-L1 Expression Through IRF7

**Figure 4:**
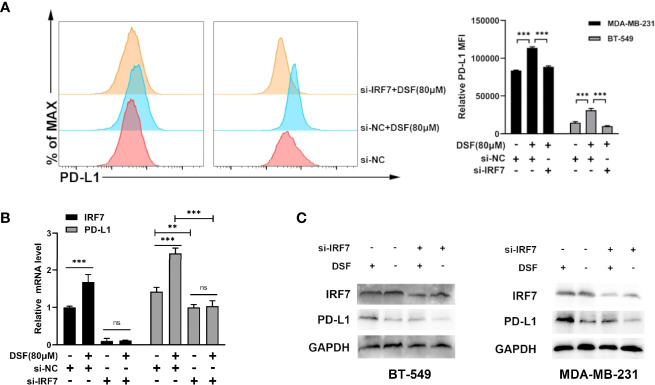
DSF regulates PD-L1 expression through IRF7. **(A)** The MDA-MB-231 cell and BT-549 cell transfected with siRNA-IRF7 were treated with DSF for 48 h, and PD-L1 expression was measured by flow cytometry analysis. Right panel, quantification of relative MFI. **(B)** The mRNA expression level of IRF7 and PD-L1 in the MDA-MB-231 cell transfected with siRNA-IRF7 or siRNA-NC after the treatment of indicated concentration of DSF for 48 h. **(C)** The protein level of IRF7 and PD-L1 in MDA-MB-231 cell and BT-549 cell treated with siRNA-IRF7 or treated with siRNA-IRF7 or siRNA-NC were measured by western blotting after treatment with indicated doses of DSF for 48 h. **p < 0.01, ***p < 0.001. ns, no statistic significance.

To investigate if DSF regulated PD-L1 through IRF7 in TNBC cell lines, we then detected whether the human BT-549 and MDA-MB-231 cells transfected with siRNA-IRF7 can significantly attenuate the upregulation of PD-L1 caused by DSF treatment in membrane protein level (**Figure 4A**). Next, the mRNA expression of PD-L1 was quantified by RT-PCR after DSF/si-RNA-IRF7 treatment. The results showed that when combined with si-RNA-IRF7, the upregulating effects of DSF on PD-L1 were significantly reduced. The level of PD-L1 transcription was significantly inhibited after knocking down IRF7 (**Figure 4B**). Western blot was applied to verify that si-RNA-IRF7 can cancel PD-L1 upregulation by DSF at the total protein level (**Figure 4C**). These results implicated that the regulation effects of DSF on PD-L1 was mediated by IRF7.

### Combination of Anti-PD-1 Therapy With DSF Improved Antitumor Activity

**Figure 5:**
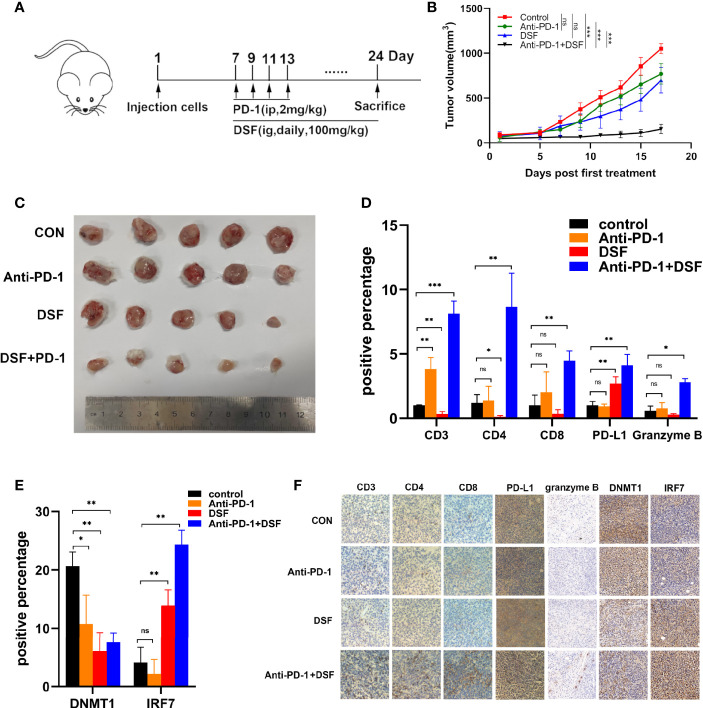
The combination of anti-PD-1 therapy with DSF improves antitumor activity. **(A)** BALB/c mice were inoculated s.c. with 4T1 cells. Seven days after inoculation, mice began to receive DSF (i.g., daily, 100 mg/kg), anti-PD-1 blocking antibodies (i.p., four times, 2 mg/kg), a combination of reagents or solvent control as indicated. **(B)** Representative photographs of 4T1 tumor of the mice after treatment with DSF and/or anti-PD-1 blocking antibody. **(C)** 4T1 implanted tumor-bearing mice were randomly enrolled in different treatment groups as indicated. For each treatment group, tumor volumes were measured every 3 days and plotted individually. **(D)** The positive percentage of CD3, CD4, CD8, PD-L1, and granzyme B in 4T1 tumors. **(E)** The positive percentage of DNMT1 and IRF7 in 4T1 tumors. **(F)** Immunohistochemical staining for CD3, CD4, CD8, PD-L1, granzyme B, DNMT1, and IRF7 in 4T1 tumors. *p < 0.05, **p < 0.01, ***p < 0.001. ns, no statistic significance.

Increasing PD-L1 expression could improve the response to PD-1 blockade therapy. Thus, we explored combined efficiency of anti-PD-1 Ab and DSF *in vivo*. The schedule of animal experiments was shown in a flowchart (**Figure 5A**). The results showed that DSF reduced the tumor burden moderately, anti-PD-1 Ab inhibited tumor growth slightly, and the co-treatment strategy exhibited higher antitumor efficacy than each treatment alone (**Figures 5B, C**). In addition, mice can tolerate this combination treatment well. Immunohistochemistry (IHC) analysis demonstrated that both PD-L1 expression was upregulated in the DSF group as well as the DSF and anti-PD-1 Ab co-treatment group, while the population of tumor-infiltrating CD8+ T cells was downregulated in the tumor tissues of mice treated with DSF alone. Furthermore, the population of granzyme B+, CD8+ in the tumor tissue was significantly increased in the combination group, suggesting that the combined treatment could improve T cell activities in mice. Taken together, these findings illustrated that DSF combined with anti-PD-1 Ab had potential therapeutic benefits (**Figures 5D, F**). Finally, we detected the expression of DNMT1 and IRF7 in mice tissues. DNMT1 was downregulated and IRF7 upregulated in the DSF group and the DSF and anti-PD-1 Ab co-treatment group, which was consistent with the results *in vitro* (**Figures 5E, F**).

### The Modulation of TIME in the Co-Combination of DSF and the Anti-PD-1 Ab

**Figure 6:**
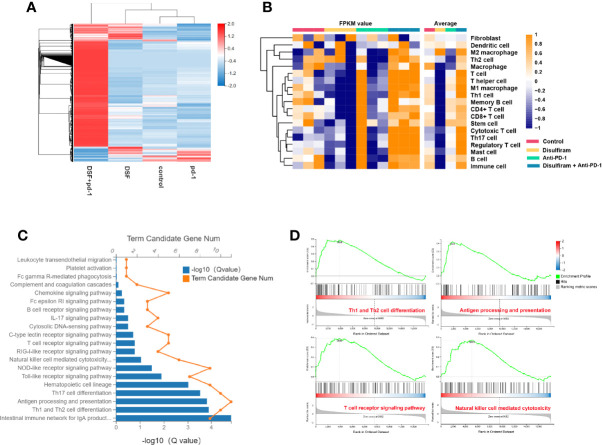
The modulation of tumor immune microenvironment in the combination of DSF and anti-PD-1 therapy. **(A)** Heat map shows gene expression profiles of a selected list of the immune microenvironment showing fold changes in the groups of control, DSF, anti-PD-1 antibody, combination. **(B)** The MCP counter algorithm was utilized to analyze the degree of infiltration of immune cells with mRNA expression in the groups of control, DSF, pd-1, combination. **(C)** The Gene ontology (GO) and Kyoto Encyclopedia of Genes and Genomes (KEGG) functional enrichment analyses of mRNAs in the groups of control *vs* DSF. **(D)** KEGG functional enrichment analysis of the anti-PD-1 blocking antibody groups and the combination of DSF and the anti-PD-1 blocking antibody groups based on GSEA.

To further explore the effects of DSF and anti-PD-1 blocking Ab co-treatment on the immune microenvironment in 4T1 mouse xenograft tumor model, the expression profiles of immune microenvironment-related genes in the four groups were analyzed by RNA-seq. Notably, the gene expression profile of DSF and anti-PD-1 Ab co-treatment group was significantly different from other three groups (**Figure 6A**). For further validation of DSF and anti-PD-1 blocking Ab co-treatment as a potential immune regulator, we measured the tendency of residential immune cells in different treatment groups by calculating the degree of immune infiltration through MCP counter in R. We found the tumors in DSF group were in a low immune activation state (**Figure 6B**). This might be due to the upregulated expression of PD-L1 by DSF in tumor cells resulted in an impaired T cell function. To further explore the potential molecular mechanism of DSF regulating tumor immune function, the GO and KEGG pathway enrichment analysis was done to reveal the inactive state of immune-associated pathways (**Figure 6C**), such as Th1 and Th2 cell differentiation (Q<0.01), as well as antigen processing and presentation (Q<0.01). We speculated that the co-treatment of DSF and anti-PD-1 Ab can overcome the immunological side effects caused by DSF. To reveal the differences in biological function between the anti-PD-1 Ab group and the DSF and anti-PD-1 Ab co-treatment group, gene set enrichment analysis (GSEA) was performed (**Figure 6D**). The results indicated that four pathways (i.e., Th1 and Th2 cell differentiation, antigen processing and presentation, natural killer cell-mediated cytotoxicity, and T cell receptor signal transduction) were significantly enriched in the combination groups, whereas no pathway was markedly enriched in anti-PD-1 blocking Ab groups (P<0.05).

## Materials and Methods

### Cell Culture

Human BC cell lines BT-549, MDA-MB-231 and mouse BC cell line 4T1 were supplied by ATCC. BT549 was cultured in RPMI 1640 (Thermo Scientific HyClone, USA), while MDA-MB-231 and 4T1 were cultured in DMEM (Thermo Scientific HyClone) containing 1% penicillin-streptomycin and 10% fetal bovine serum (FBS), followed by incubation in an atmosphere of 5% CO2 at 37°C.

### Reagents and Abs

DSF was obtained from Sigma (USA) and then dissolved in DMSO. siRNA of IRF7 (5′-TCGAGTGCTTCCTTATGGA- 3′) (RiboBio, Guangzhou, China). Anti-DNMT1, IRF7, PD-L1, granzyme B, CD3, CD4, CD8, and GAPDH Abs were obtains from ABclonal (Wuhan, China). PE-conjugated PD-L1 Ab were obtained from BioLegend (San Diego, CA, USA). The DNMT Activity/Inhibition Assay Ultra Kit (Colorimetric) was purchased from EpiGentek (USA). The anti-PD-1 blocking Ab were obtained from Innovent Biologics (China).

### Immunoblotting

The cells were rinsed twice with pre-cold PBS and lysed in RIPA Buffer for 15 min, followed by centrifugation (15,000 rpm, 15 min, 4°C). Total protein content was measured using the BCA kit (Beyotime). After separation on 10% SDSPAGE, the samples were transferred onto a PVDF membrane. Subsequently, the membrane was inhibited with 5% skimmed milk in 0.1% TBST for 1 h, and then exposed to primary Abs at 4°C. On the next day, the membranes were rinsed thrice with TBST for 15 min and exposed to the corresponding secondary Ab (1:4,000 dilution, Servicebio, Wuhan, China) for 1 h. After washing, the protein blots were detected using a SuperSignal West Pico Chemiluminescent Substrate (Pierce, USA). GAPDH was employed as an internal control for normalization.

### RT-PCR

Total RNA was extracted with Total RNA Kit I (Omega, USA). cDNA synthesis was conducted with SYBR^®^ Premix Ex TaqTM II (Takara Bio, Japan) by following the manufacturer’s protocols. RT-PCR was performed on a Step One Plus RT-PCR system (Applied Biosystems) using SYBR Green Mastermix (Takara Bio). The sequences of the primers used are as follows: DNMT1 F: 5ʹ 5-CGGCAGACCATCAGGCATTCTAC-3ʹ and R: 5ʹ-CACACCTCACAGACGCCACATC-3ʹ; IRF7 F: 5ʹ-CTCCTTGGAGAGATCAGCAG-3ʹ, and R: 5ʹ-CAGCGG-AAGTTGGTTTTCC-3ʹ; PD-L1 F: 5ʹ-GCTGCACTAATTGTCTATTG-GG-3ʹ and R: 5ʹ-CACAGTAATTCGCTTGTAGTCG-3ʹ; GAPDH F: 5ʹ-ACCACAGTCCATGCCATCAC-3ʹ and R: 5ʹ-TCCACCACCCTGTTGCTGTA-3ʹ.

### Flow Cytometric Analysis

The harvested cells were incubated with PE-conjugated PD-L1 Ab (BioLegend) for 30 min at 4°C, rinsed twice with 4°C PBS, evaluated with flow cytometry, and analyzed by FACSDiva Software (BD Bioscience, NJ, USA).

### Bisulfite Sequencing PCR

MDA-MB-231 and BT-549 cells (1×10^5^/wells) were grown on 6 cm dishes for 24 h, and then exposed to vehicle or the corresponding drugs for 48 h. Then, genomic DNA was extracted, followed by bisulfite treatment using the Qiagen EpiTect kit. PCR cycle (98°C for 30 s; followed by 35 cycles of 98°C for 10 s, 60°C for 30 s, and 72°C for 10 s; 72°C for 2 min; 4°C hold) was conducted with Q5 Hot Start High-Fidelity Master Mix (NEB). Methyl Primer Express™ v1.0 (ThermoFisher) was used to design primer sequences for IRF7 promoter region (F: 5′-TTGGGTTGTAGTGGAGTGGTTTTATT-3′; R: 5′-CATCTCTCAAACTCCCCCAACTCTT-3′). The PCR products were detected through electrophoresis and purified by Gel and PCR Clean-up System (Promega). After purification, Zero Blunt™ TOPO™ PCR Cloning Kit (Invitrogen) was used to insert the products into pCR™4Blunt-TOPO^®^ Vector. Lastly, Sanger sequencing (Sangon) was carried out.

### Data Collection and Methylation and Copy Number Variation Analysis

The gene expression RNAseq data (1,104 tumor tissues and 113 normal tissues) of the cohort: Genomic Data Commons Cancer Genome Atlas-Breast Cancer (GDC TCGA-BRCA) were obtained from the https://xenabrowser.net/datapages/. Total BC specimens were ordered according to methylation score levels. The beta-value cutoff ranges for hypermethylation and hypomethylation were 0.7–0.5 and 0.3–0.25, respectively (35, 36). R 3.6.0 was used to analyze the relationship between the level of IRF7 methylation and transcription. MEXPRESS is an easy-to-use tool for visualizing gene expression, DNA methylation, clinical TCGA data, and the relationship among them (37). Given the important effects of methylation and CNVs on gene expression, MEXPRESS was used to explore the association between IRF7 expression and methylation/CNVs.

### Gene Correlation and Gene Expression Analysis

GEPIA database (http://gepia.cancer-pku.cn/index.html) was employed to verify the significant relation between IRF7 and PD-L1, and the Pearson correlation was used to analyze the correlation between the two genes. The gene transcript expression in TNBC/basal-like compared with normal breast cases in TCGA and GTEx data cohorts by GEPIA2 (http://gepia2.cancer-pku.cn/). The p-value were set as 0.01.

### siRNA Transfection

siRNA targeting IRF7 was supplied by RiboBio (China). After transfection with siRNA (50 nM), the cells were analyzed by GenMute transfection reagent (SL100568; SignaGen Laboratories, China) as per the manufacturer’s protocol.

### Animal Experiments

BALB/c mice (4–6 weeks old, female) were procured from Beijing HFK Bioscience (China) and maintained under SPF conditions in accordance with the animal care guidelines of Huazhong University of Science and Technology (HUST). The experimental protocol was approved by the Ethical Committee of HUST. 4T1 cells (1 × 10^6^) were subcutaneously transplanted into the right flank of mice. The tumor-bearing mice were randomly categorized into four groups (n = 5/group): control; anti-PD-1 blocking Ab i.p.; DSF p.o; anti-PD-1 blocking Ab + DSF. Tumor dimension was assessed by vernier caliper, and tumor volume was calculated as follows: 0.5×width^2^×length. After 28 days, the mice were sacrificed, and the subcutaneous tumors was isolated, recorded, and subsequently analyzed. DSF (100 mg/kg) was administered daily *via* the p.o. route starting from day 7 after tumor implantation and continuously for 3 weeks. Anti-PD-1 blocking Ab (200 µg; BE101, BioXCell) was administered i.p. every other day for four times starting from day 7 after tumor implantation. The mice in control group were treated with solvent (5% Tween 80, 30% PEG300, and 2% DMSO). Tumor size was recorded using a digital caliper every 3 days and calculated as follows: 0.5×width^2^×length.

### Staining of Tumor Tissue Sample

4T1 xenografted tumors in BALB/c mice were formalin-fixed and embedded in paraffin. Briefly, after deparaffinization, rehydration, and antigen retrieval, tissue sections were exposed to primary antibody (DNMT1, IRF7, PD-L1, granzyme B, CD3, CD4, or CD8). Then, the sections were exposed to biotinylated goat-anti-mouse IgG secondary antibody and streptavidin-conjugated HRP, and finally developed with 3,3′-diaminobenzidine (DAB). Images were collected using a high-resolution slide scanning system (3DHISTECH Ltd, Pannoramic MIDI). Image-pro Plus 6.0 software (Media Cybernetics, Inc., Rockville, MD, USA) was used to select the same brown color as the unified standard for judging the positivity of all photos, and the positive area of each photo was obtained by analyzing each photo.

### RNA Deep Sequencing

Total RNA was isolated from the 4T1 xenografted tumors tissue sample using TRIzol Reagent (Invitrogen, USA), and then subjected to RNA deep sequencing with MGISEQ2000 platform at Beijing Genomics Institute (BGI, China). The obtained sequencing reads were expressed as the FPKM (fragments per kilobase of exon per million reads) for each transcript. KEGG and single sample GSEA (ssGSEA) analyses were described in the previous study (38). The R-3.4.3 software tools were used to perform bioinformatics analysis and generate figures.

### Statistical Analysis

The data were shown as mean ± S.E. Statistical tests were performed by GraphPad Prism v8.0 and R language v3.4.3. Unpaired Student’s t-test (n<30) and Wilcoxon test (n>30) were used to compare the difference between two groups. Level of significance was set as p<0.05. The Pearson correlation was used to analyze the correlation between the two genes.

## Discussion

**Figure 7:**
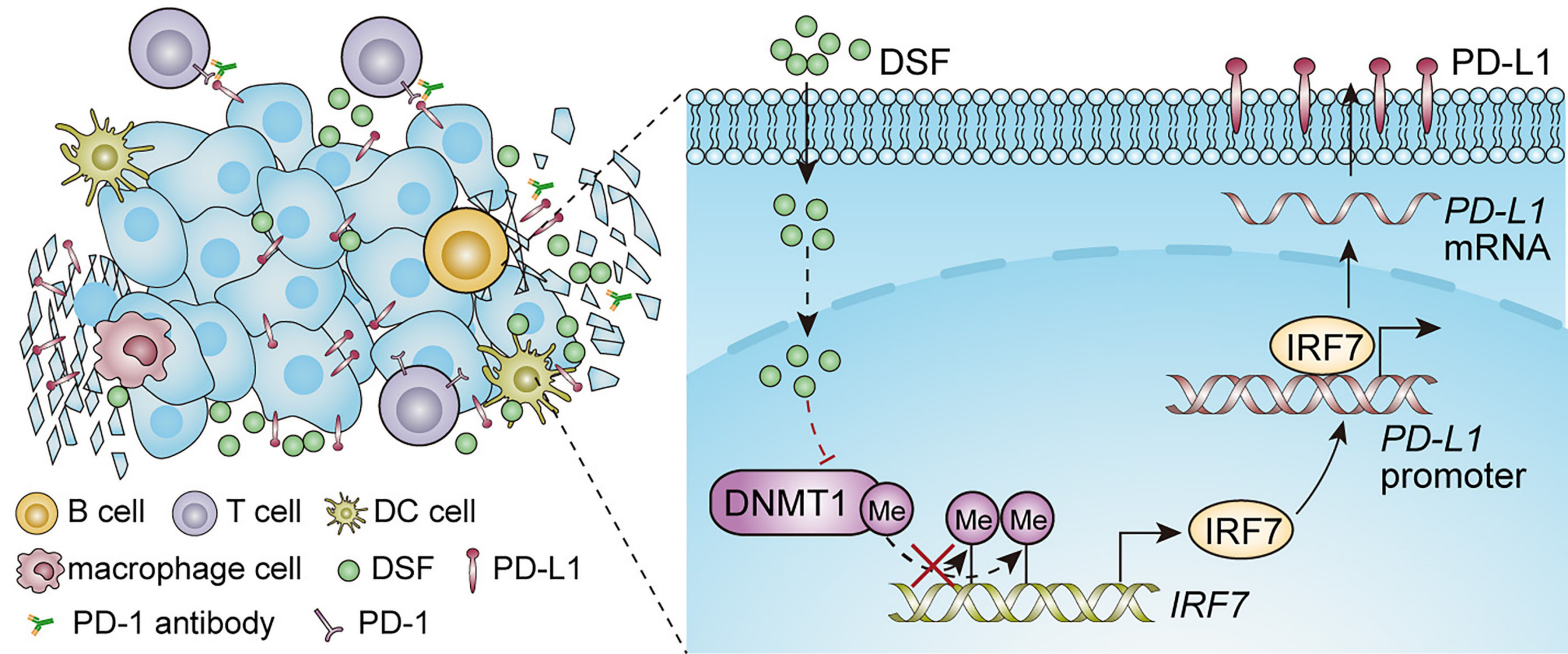
An illustration of the proposed working model. Synergistic effect of DSF and PD-1 blocking antibody in the treatment of triple negative breast cancer.

Despite anti-PD-1 therapy has shown promising clinical benefits in patients with TNBC, a significant fraction of patients remains unresponsive to this therapy. The key to improve the anti-PD-1 therapy is the formation of combination therapies (39). Accumulating evidences have indicated that altered PD-L1 expression by small molecules can modify the efficacy of anti-PD-1 therapy in preclinical phase (40–42). In this work, as **Figure 7** shows, we found that DSF could upregulate PD-L1 expression through hypomethylating IRF7 *via* inhibition of DNMT1 activity and expression, and improve the anti-PD-1 therapy by modulating TIME. Although increased PD-L1 expression represents an immunosuppression status, DSF combined with anti-PD-1 Ab could overcome it.

IRF7 is an important regulator of type I IFN responses, and its transactivation can amplify the generation of type I IFNs *via* a positive feedback loop (43). IRF7 was found by Bidwell et al. to be overexpressed in NBT and primary BC tissue, while much lower expressed in bone metastasis (23). Restoration of IRF7 inhibited bone metastasis of BC cells by inducing the production of type I IFNs, and caused an increasing of CD8+ T cells in the blood samples of 4T1 tumor-bearing mice. Lan et al. reported that the upregulated expression of IRF7 in doxorubicin- and methotrexate-treated tumor cells could induce a switching from the myeloid-derived suppressor cell-mediated immune responses to the CD4+/CD8+ T cell-dependent anticancer responses through constitutive activation of type I IFN pathway (22). However, IRF7 is also reported to enhance constitutive PD-L1 expression, independent of IFN induce, resulting in decreasing CD8+ TIL expansion (21). IRF7 has the potential to exert multifaced effects on regulating TME (44). The expression of IRF7 can be epigenetically regulated by DNA methylation (45). DNMT inhibitors, decitabine and azacytidine, can induce the DNA hypomethylation of IRF promoter leading to transcriptional activation of IRF7 (46). In our study, DSF acted as a DNMT1 inhibitor and upregulated IRF7 expression by DNA hypomethylation, whose effect is similar to other DNMT inhibitors.

PD-L1 expression is one of the biomarkers to help select patients for anti-PD-1 therapy. Increasing evidence has shown that abnormal PD-L1 expression can affect the efficacy of anti-PD-1 therapy (42, 47). PD-L1 expression can be regulated by transcriptional control. Transcriptional factors, such as MYC, BRD4 and IRF1, participate in the activation of PD-L1 by binding to its promoter. IRF7 could also directly bind to PD-L1 promoter and enhance its transcriptional expression (9). Our study, like many other literatures, showed a positive correlation between PD-L1 and IRF7 at both transcriptional and protein levels in TNBC (48, 49). However, Chang and colleagues showed that PD-L1 protein level is negatively correlated with IRF7 in lung squamous cell cancer tissues, which may result from the effect of PD-1/PD-L1 reverse signaling on eIF2α/ATF4 activation with subsequent downregulation of IRF7 expression (50). DSF-induced PD-L1 expression was abolished by IRF7 knockdown in our study, suggesting that DSF upregulated PD-L1 expression *via* IRF7 restoration. Additionally, although DSF epigenetically restoration of IRF7, immunological suppressive TME was observed in tumors treated by DSF (**Figure 6**). We speculate the reason might be constitutively expression of PD-L1, which was mediated by IRF7 directly promoting transcription of PD-L1, leading to abrogating CD8+ TIL expansion (21).

DSF, an old anti-alcohol drug, has been shown to possess anticancer effects on various malignancies for many years. However, recent study reported that DSF combined with copper (DSF/Cu2+) could inhibit tumor proliferation in immunodeficient mice but failed in immunocompetent mice (45). Zhou and colleagues further found DSF combined with copper (DSF/Cu2+) was reported to upregulate PD-L1 expression by suppressing PARP1/GSK3β in hepatocellular carcinoma cells and ultimately prevented CD8+ TIL infiltration (51). However, co-treatment of DSF/Cu2+ and anti-PD-1 Ab improved antitumor immunity in mice and showed better antitumor activity than the monotherapy. Terashima et al. demonstrated that DSF inhibited FROUNT and suppressed macrophage deposition and its tumor-promoting potential (29). In our study, co-treatment of DSF and anti-PD-1 Ab noticeably elevated the population of granzyme B-positive CD8+ TIL and synergistically inhibited tumor growth compared to monotherapy. In contrast, anti-PD-1 Ab alone showed no growth inhibitory effect on 4T1 tumors with low CD8+ TIL, which is concordant with other studies (52).

Concordant with other studies, we found DSF treatment alone did not exert antitumor effects along with upregulated PD-L1 expression as well as a decreased immune cell infiltration in immunocompetent mice model, and we also observed that co-treatment of DSF and anti-PD-1 Ab improved the efficiency of anticancer with increased CD8+ TIL and activated antitumor immune pathway (29, 51). Furthermore, our RNA-seq results indicated that the combination of DSF and anti-PD-1 Ab reversed the immunological suppressive effects caused by DSF, and four pathways (i.e., Th1 and Th2 cell differentiation, antigen processing and presentation, natural killer cell-mediated cytotoxicity, and T cell receptor signal transduction) were significantly enriched in the combination groups, whereas no pathway was markedly enriched in anti-PD-1 blocking Ab groups.

A large number of studies have proved that DSF has superior anti-BC effects *in vitro* and *in vivo*, providing a new potential direction for TNBC treatment (53–55). A phase II trial of copper and DSF against metastatic BC (NCT03323346) was carried out to provide clinical evidence for introducing this novel combination therapy to metastatic BC patients who had failed conventional systemic or locoregional therapy. Our results indicated that the supplement of DSF to the anti-PD-1 therapy could improve the therapeutic efficacy than the monotherapy.

In conclusion, our findings demonstrate that DSF upregulates PD-L1 expression *via* DNMT1-mediated IRF7 hypomethylation and restoration in TNBC, and enhances the antitumor potential of anti-PD-1 Ab by modulating TME in **Figure 7**. DSF combined with anti-PD-1 Ab could serve as a novel option for relapse or metastatic TNBC.

## Data Availability Statement

The datasets presented in this study can be found in online repositories. The names of the repository/repositories and accession number(s) can be found below: (https://www.ncbi.nlm.nih.gov/gds/), GSE186885.

## Ethics Statement

The animal study was reviewed and approved by Ethical Committee of Huazhong University of Science and Technology.

## Author Contributions

LZ and GW designed the experiments. XZ, ZL, MM, and QW performed experiments, data collection, and analysis. XZ and LZ analyzed data and drafted the manuscript. All authors contributed to the article and approved the submitted version.

## Funding

This work was supported by grant from the National Natural Science Foundation of China (no. 81672940) and the Clinical Research Physician Program of Tongji Medical College, Huazhong University of Science and Technology (no. 5001530053).

## Conflict of Interest

The authors declare that the research was conducted in the absence of any commercial or financial relationships that could be construed as a potential conflict of interest.

## Publisher’s Note

All claims expressed in this article are solely those of the authors and do not necessarily represent those of their affiliated organizations, or those of the publisher, the editors and the reviewers. Any product that may be evaluated in this article, or claim that may be made by its manufacturer, is not guaranteed or endorsed by the publisher.
